# 

**Published:** 1885-08

**Authors:** 


					﻿ia/cts.\acppy.
AUGUST, 1885.
$1.00 per year.
WALES*
eJov^N/XLf
HEALT El
ESTABLISHED 18^4
Vi-3 2
#2.8
OFFICE, 75 & 77 BARCLAY STREET, NEW YORK,
Entered as Second-class Matter at the Post-Office, New York.
				

